# Haptic Glove TV Device for People with Visual Impairment

**DOI:** 10.3390/s21072325

**Published:** 2021-03-26

**Authors:** Diego Villamarín, José Manuel Menéndez

**Affiliations:** 1 Grupo de Aplicación en Telecomunicaciones Visuales, Universidad Politécnica de Madrid, 28040 Madrid, Spain; jmm@gatv.ssr.upm.es; 2 Departamento de Eléctrica, Electrónica y Telecomunicaciones, Universidad de las Fuerzas Armadas ESPE, 170501 Sangolquí, Ecuador

**Keywords:** haptic glove, immersive TV, social TV, accessible TV, DTT, interactive TV

## Abstract

Immersive video is changing the way we enjoy TV. It is no longer just about receiving sequential images with audio, but also playing with other human senses through smells, vibrations of movement, 3D audio, feeling water, wind, heat, and other emotions that can be experienced through all human senses. This work aims to validate the usefulness of an immersive and interactive solution for people with severe visual impairment by developing a haptic glove that allows receiving signals and generating vibrations in hand, informing about what happens in a scene. The study case presented here shows how the haptic device can take the information about the ball’s location in the playing field, synchronized with the video reception, and deliver it to the user in the form of vibrations during the re-transmission of a soccer match. In this way, we take visually impaired people to live a new sensory experience, allowing digital and social inclusion and accessibility to audiovisual technologies that they could not enjoy before. This work shows the methodology used for the design, implementation, and results evaluation. Usability tests were carried out with fifteen visually impaired people who used the haptic device to attend a soccer match synchronized with the glove’s vibrations.

## 1. Introduction

Nowadays, technology on television and in all audiovisual resources has evolved to offer a high image and sound quality and other sensations and emotions. Interactivity with users has become crucial since now there is the possibility of having content that makes the viewer participate in the programming. Furthermore, viewers have evolved into users who no longer just watch a series of sequential images but have active participation in the programming interacting through movement, sensory sensations, changing or deciding the film’s direction, choosing options, participating in surveys, making purchases on TV, etc. [1,2,3,4].

This work’s objective was born analyzing several current television concepts, one of them being immersive TV. There are movies and movie theaters that call themselves 4D. Apart from the typical sequence of images and audios, they include some sensorial options, such as movements and vibrations from unique chairs. The wind is activated with big fans, essences that come out of the chairs, water through sprinklers, heat, among others that are synchronized with the screen’s events [5,6,7,8,9].

Another concept that was studied is the issue of accessibility to television and multimedia video content. Several works have already been implemented to make audiovisual content accessible to people with some sensory impairment. For example, there is audio description for visually impaired people, subtitles, Teletext and even sign language for people with hearing impairments [10,11,12,13,14]. Some works combine accessibility in TV with virtual reality (VR) and Multi-Screen TV Experiences taking it to different applications such as culture and sports [15,16]. These works have not been widely exploited yet and, as they are not mandatory in all TV broadcasts, the issue of digital inclusion for people with impairments is still a problem.

Social TV also came into the analysis because it has changed lately. Before, TV was linear, and there was only one broadcast that you could pick up on your TV tuner. However, with the exponential growth of data networks, video consumption has returned to on-demand. You can also have multiview channels or multiscreen videos [17,18].

Especially on Social TV sports, the advancement of social networking technologies has changed some sports fans’ viewing experience. Now viewers can interact with other fans in real-time through social network sites while watching a televised sporting event such as the world soccer championship like show the work of Hwang et al. [19].

Over-the-top (OTT) networks offer various video content with trendy platforms such as Netflix, Youtube, Amazon Video, Vimeo, among others, that the user can access anywhere, with any device any time. The growth of social networks also plays an essential role since these networks allow the participation and interaction between thousands of users who are attending video content, either through TV or through other multimedia devices connected to an IP data network [20,21,22].

Augmented Broadcasting (AB) service tries to provide personalized broadcasting service over a TV program. Every viewer may watch personalized augmented content (AC) overlaid on the same TV program. The paper of Ha et al. [23] introduces prototypes of user interface (UI) devices for AB services. They made devices like smart pads and haptic gloves. The authors of this paper discussed their UI for AB service prototypes. Their evaluation shows AB services make TV programs more helpful in an interactive exchange of user responses and additional information.

While analyzing these technologies, a problem that has not been fully answered yet becomes apparent. According to the World Health Organization (WHO), 1300 million people live with some form of visual impairment, of which 188.5 million people have a moderate visual impairment, 217 million have a moderate to severe visual impairment, and 36 million are blind [24]. Most of them consume TV content, but when they attend TV programming, they face several problems. Mainly, most of the content they find available is not accessible for them to fully understand what is happening in a scene. Most of the time, these people can only listen to the characters’ dialogues or, if they are lucky enough, have a commentator or storyteller to describe what is happening in the scene. This has been solved partially by adding audio descriptions to the original audiovisual content. However, only a tiny percentage of films and documentaries have it. It is not easily accessible because it is not available on most Digital Terrestrial TV (DTT) but instead in paid audiovisual content. Nonetheless, the audio description does not entirely solve this problem since many details are still lost. In some cases, it is almost impossible to have audio descriptions, such as in sports events, news, live programs, among others [25].

In the TV sports broadcast (whether soccer, football, rugby, basketball, handball, tennis, among others.), people with an audiovisual impairment only have access to audio. One could say that their eyes are the narrative of the sports reporter or commentator who is describing what is happening at the sports meeting. However, following the narrative, they only manage to perceive a part of the whole amount of details in a match. It would be practically impossible for any narrator to describe all the details of what is happening in a game every time.

As a case for this work, we are using soccer, one of the most popular sports globally, as an example. In this case, the main problem that visually impaired people have is not knowing the ball’s location within the field unless the narrator says it very explicitly. When talking to these people through personal interviews, it was possible to identify whether it is a problem for them to follow a sporting event on TV since they lose all the matches when they do not have the narrator’s voice.

It is worth mentioning that understanding what happens in a sports scene is not only present when they attend a sports meeting on television or video stream. They also have it when they listen to a sports narration on the radio and even have it when visually impaired people attend a sports meeting as fans in a live sports scenario.

In that regard, this work aims to develop a way to take the information about the ball’s location during a sports match and deliver it to people with visual impairments. This will help visually impaired people’s digital inclusion, producing an immersive, accessible, and social system that allows them to participate more actively in the programming and have a better interaction with the rest of the audience.

Haptic devices are mechanical devices that allow to receive and deliver information by touch. Right now, there is an emerging boom in the construction of haptic devices for many different purposes. Since blind people cannot receive visual information, their audible information is incomplete and deficient. The use of haptic devices to deliver more immersive sensory information could become an ideal complement to produce a more inclusive experience for the visually impaired people who find it difficult to attend a sporting event.

Haptic technology can take and present information in many different ways: generating a sensation of high relief, generating vibrations through unique skinsuit fabrics, generating movements through mechanical arms, among others. This work seemed feasible to design and build a haptic glove with motors located in strategic positions. In this way, it will be able to generate vibrations coded to deliver information about the location of, for example, the position of a tennis player on the court or, in this study case, the position of the ball within the soccer field.

There are currently various companies in the industry that develop haptic technologies, especially gloves focused on virtual reality, in which they occupy a variety of sensors and actuators. They present several solution kits that include development software [26,27]. Others even go beyond this and present wearable technologies where actuators can be embedded in the fabric to add haptic sensations into clothing for customizable user experiences, making them more real and interactive [28,29]

From the evaluation done on these products, we decided not to opt for one of these solutions but to build our glove with haptic feedback vibratory discs, delivering haptic-tactile vibratory stimuli wholly adapted to our objective. The system must be easy to learn and interpret and must not be invasive so that blind people like to use the device and give them an enjoyable and immersive user experience. We also want the system to be moderately priced and affordable for blind users.

The major motivation for this work’s development is undoubted to provide a technological solution that makes them feel that TV can be accessible and inclusive regardless of their visual impairment. In one of the interviews we had with blind people, one of them gave us a very simple but shivery challenge: when you go to a sporting event, blindfold yourself for a few minutes, and you will feel exactly what we feel. It is easy to infer that if they do not have a sighted person to narrate the scene taking place in a sporting event or any activity, the blind person is totally lost off the space. That also happens on TV when the content does not have sound with the right narrative. This simple exercise gave us the necessary push to work on this solution with great pleasure.

The related works with haptic in immersive telecommunication and other interesting applications are detailed in Section 2. The proposed methodology for designing the haptic glove, the diagrams, and a general explanation of its operation are detailed in Section 3 of this work. Section 4 shows the development and implementation of the haptic glove. Section 5 shows how to achieve the integration between the glove and the TV. Section 6 shows the analysis of the results according to the evaluation with visually impaired people who used the glove. Finally, the discussion and the proposal for future work are included in Section 7 and Section 8 respectively.

## 2. Related Works

Several previous related works have explored haptics in the context of multimedia systems. The work of Reiner et al. [30] explores the role of haptics in immersive telecommunication applications and analyzes the contribution of haptics to the sense of presence in various applications. On the books of Engineering haptic devices [31,32], concrete technical aspects of haptic manipulation and manipulators are considered beginning with higher-level subjects like control and kinematics and proceeding with a detailed discussion of actuators and sensors and the interfaces to and from the mechanical environment.

Current computer technologies rely on audiovisual information to establish an information interface between humans and machines. The work of [33] present a validation of Haptic Enabling Technology (HET), which transmits information and senses of touch and power, attracted by the desire for more realistic experience. They examined the factors affecting HET-based products’ adoption by developing an integrated research framework that combines Innovation and Diffusion Theory (IDT) and Technology Acceptance Model (TAM).

There is an excellent review paper presented by Danieau et al. [34]. They speak about haptic technology has been widely employed in applications ranging from teleoperation and medical simulation to art and design, including entertainment, flight simulation, and virtual reality. They mentioned that today there is a growing interest among researchers in integrating haptic feedback into audiovisual systems. A new medium emerges from this effort: haptic-audiovisual (HAV) content. They presented the techniques, formalisms, and critical results pertinent to this medium. Moreover, they showed three main stages of the HAV workflow: the production, distribution, and rendering of haptic effects.

Lee et al. [35] present in your work a vibrotactile display system designed to provide an immersive live sports experience, to compensate for visually confusing ball movements in a soccer match.

Rehman et al. [36] proposed a new method of rendering live football games on mobile phones using vibration. A mobile phone is synchronized with the ball in the whole field. By holding the phone, users can experience dynamic movements of the ball, know attacking directions, and which team is leading the attack.

Kim et al. [37] describes a tactile system designed to provide viewers with passive, on-skin sensations synchronized with audiovisual media.

The three works mentioned above present related ideas to our proposal, but they have a different approach and were not focused on being a solution for blind people. They used different and old device technologies.

Junior et al. [38] present a Tactile Glove Device proposal, and it is also similar to our haptic glove TV device. However, this work aims to develop a tactile glove device and a virtual environment inserted in the context on the internet next generation called “Tactile Internet”. Neela Harish [39] developed an Intelligent experimental glove for visually impaired people using a haptic feedback system with a Braille pad. Here they show a Global System for Mobile Communications (GSM) module that converts text inputs into Braille patterns through vibrations using DC cylindrical motors with a PIC microcontroller as the master. Therefore the words corresponding to the vibration will be intimated through the gloves to the visually impaired people.

Feintuch et al. [40] discuss the potential contribution of adding haptic feedback to VR applications in the field of rehabilitation. Theoretical foundations, as well as converging empirical evidence. They suggest that haptic feedback may enhance clinical intervention. The proposed system aims to integrate simple vibrating feedback into a video capture system, thereby producing an intervention tool of greater power and flexibility.

Bortone et al. [41,42,43,44] show a series of works related to serious games and wearable haptic devices for rehabilitation. They have several scenarios Immersive Virtual Environments (VE) with Head Mounted Display (HMD) devices and Wearable Haptic Devices for the rehabilitation of upper limb in children with Cerebral Palsy (CP) and Developmental Dyspraxia (DD). In this way, we also found interesting the work of Leonardis et al., where a novel wearable haptic device for modulating contact forces at the fingertip is presented [45].

El-Far, Eid, et al. [46,47] both works propose an XML-based description language, namely Haptic Application Meta Language (HAML). They describe HAML as a technology-neutral of haptic models. It contains specifications and ergonomic requirements for haptic hardware and software interactions. Their goal is to allow the creation of plug-and-play environments in which a wide array of supported haptic devices can be used in a multitude of virtual environments. As per their implementation, the MPEG-7 standard has been used to instantiate HAML schema by using description Schemes (DS).

Kim et al. [48] present an example of constructing a haptic-enabled broadcasting system based on the MPEG-V standard. They proposed a haptic-enabled broadcasting system’s construction processes. Their work illustrates the system data flow, from the creation of haptic contents to the rendering of these contents to the end-user, and explains a method of building the system with the MPEG-V standard. Their constructed haptic-enabled broadcasting system allows users to have more immersive interaction with the synthesized haptic multimedia, which is closely synchronized with audio-visual data.

Finally refer to AI and computer vision works, Thomas et al. [49] discusses a selection of current commercial applications that use computer vision for sports analysis, and highlights some of the topics that are currently being addressed in the research community. Kamble et al. [50] present a novel deep learning approach for 2D ball detection and tracking (DLBT) in soccer videos, posing various challenges.

## 3. Materials and Methods

To build a device that is appropriate and will fulfill the necessities of the visually impaired, the characteristics of the glove and how it will operate were designed with the help of a person who liked soccer and who had lost his sight. Several ideas about the glove’s operation were proposed to him and were validated by him based on the criteria of usability and ergonomics. After the analysis, we also defined how the hardware and software would be used.

### 3.1. Functional Diagram

**Figure 1:**
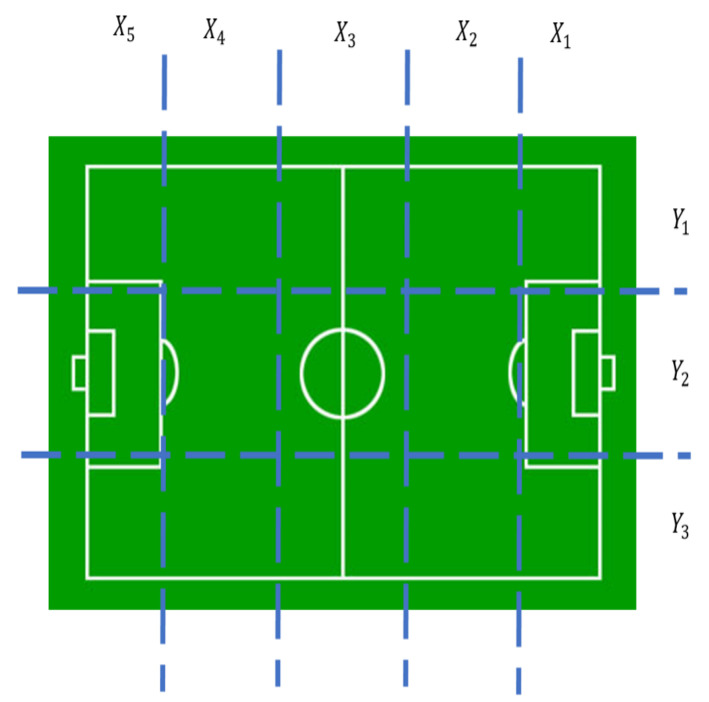
Divisions of the soccer field in the *X* and *Y* axis.

**Figure 2:**
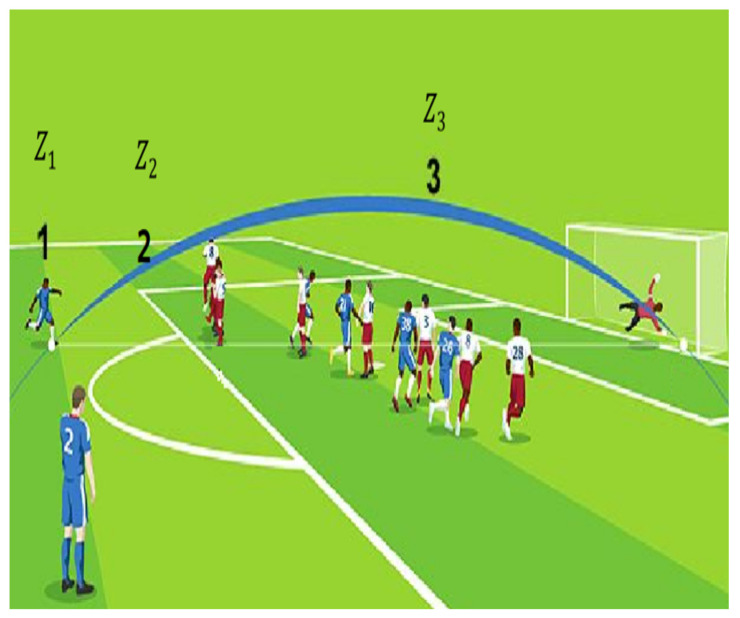
Height of the ball on the vertical axis *Z*.

The haptic glove is a device that allows the geolocation of the soccer ball within the field. To achieve that, the soccer field was divided into small regions. There are five divisions in the *X*-axis (length of the field) and three divisions in the *Y*-axis (width of the field). This forms 15 small areas within the field. For the vertical *Z*-axis there are also three divisions (height above the court) that will denote the ball’s height within the court. Figure 1 and Figure 2 denote the divisions of the court.

Figure 1 shows the 15 regions formed by the divisions on the *X* and *Y* axis of the soccer field. For example, when the ball is in the center region of the field, it will be located in the vector $X_{3}Y_{2}$. In Figure 2 it can be seen that there are also three divisions to indicate the height of the ball above the level of the court: $Z_{1}$ when the ball is on the floor, $Z_{2}$ when the ball is at a medium height, as a reference up to the head of the players, and $Z_{3}$ when the ball is at a height that has exceeded the player’s height. For example, when a match is going to start, the ball will be located in the center of the field in the vector $X_{3}Y_{2}Z_{1}$, when a corner shot is going to be charged the ball can be in $X_{1}Y_{1}Z_{1}$ or $X_{1}Y_{3}Z_{1}$ or $X_{5}Y_{1}Z_{1}$ or $X_{5}Y_{3}Z_{1}$.

**Figure 3:**
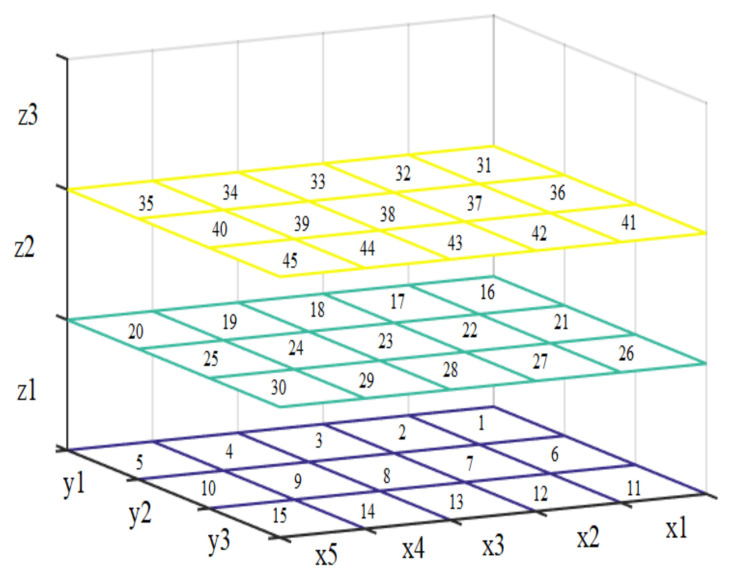
Regions formed on the soccer field for the location of the ball.

In this way, 45 positions or regions were vectored as shown in Figure 3. These locations were then represented by means of vibrations using the engines of the haptic glove.

**Figure 4:**
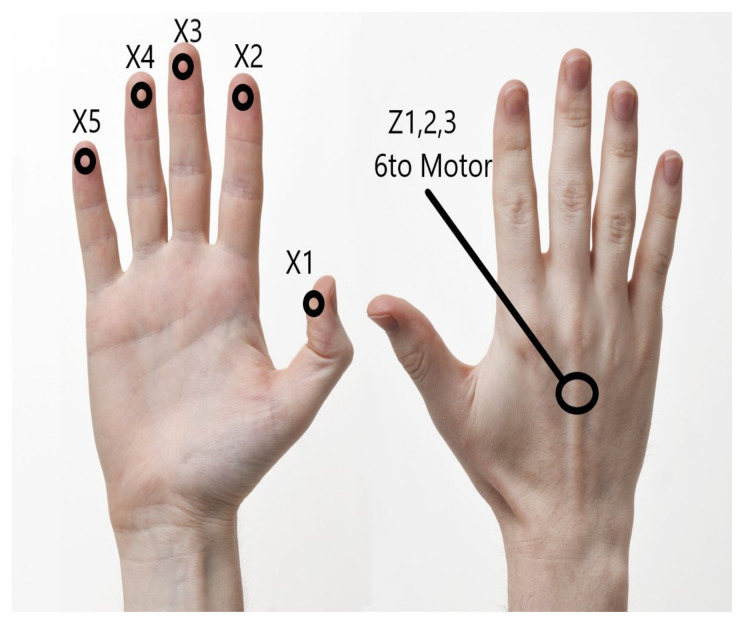
Distribution of haptic motors in the hand.

Since representing these 45 possible locations of the ball on the court using 45 different motors was not an efficient or possible option. We decided that these locations would be represented by six motors: five of them located in the yolks of each finger and one of them located in the back of the right hand, as can be seen in Figure 4.

The thumb represents the vector $X_{1}$, the index finger $X_{2}$, the middle finger $X_{3}$, the ring finger $X_{4}$, the little finger $X_{5}$. The sixth motor located on the back of the hand represents the *Z* axis (the height of the ball in the court) using three levels of vibration: $Z_{1}$ zero vibration, $Z_{2}$ medium vibration and $Z_{3}$ intense vibration. Similar to the *Z* vector, the three levels in the Y vector are represented with three levels of vibration: $Y_{1}$ low vibration, $Y_{2}$ medium vibration, and $Y_{3}$ high vibration. The motors’ vibration levels are generated by the action of haptic controllers, which are capable of generating various vibratory effects. A summary of each motor’s location in the right hand and what each vibration represents is shown in Table 1.

To explain the glove operation to visually impaired people, they are told to assume that they are in the stadium grandstand watching a soccer match with each finger on their right hand representing a location along the field. The thumb represents the area of the A team’s soccer goal. The little finger represents the area of the B team’s goal, the middle finger represents the midfield sector, and the other two fingers represent the A and B sectors teams, respectively. For the Y vector, they are told that the closer the ball is to their location in the grandstand, they will feel more intense vibration. As the ball moves away from its location, the vibration will be lowered reaching a medium level of vibration in the midfield. Likewise, regarding the ball’s height, when the ball is on ground level or a few inches from the grass, there will be no vibration. When the ball is raised to the height of the players’ heads, there will be a medium level of vibration. Moreover, when the ball is raised above the players’ heads’ height, there will be a high vibration level.

### 3.2. Sampling

Initially, the sampling of the ball location was proposed to occur every 100 ms, however during the glove testing stage, it was changed to 500 ms, that is, two samples every second. This change was done since having too many samples per second made it impossible for the user to assimilate and process all the information regarding the ball’s position. In fact, this many vibrations would be felt more like a hand massage than providing an immersive experience. With sampling, every 500 ms the results were better, but it was still necessary to reconsider this operation since it continued to be very intense to receive vibrations every half second. Thus, it was decided only to have vibrations when the ball moves from one sector to another in the soccer field. So if the game is played on the midfield (sector $X_{3}Y_{2}Z_{1}$), there will only be one vibration on the middle finger. Moreover, when the ball moves to a new sector, for example, sector $X_{4}Y_{2}Z_{1}$, then a medium intensity vibration will be felt on the ring finger. This makes the vibration frequency much lower and much easier for the user to process. It also means that the device’s power consumption is low, and therefore the battery life is extended considerably. The sampling to achieve this process was done manually (since automating it is outside the scope of this work) with a video demo of a 90-min soccer match.

### 3.3. Materials and Operation Diagram

**Figure 5:**
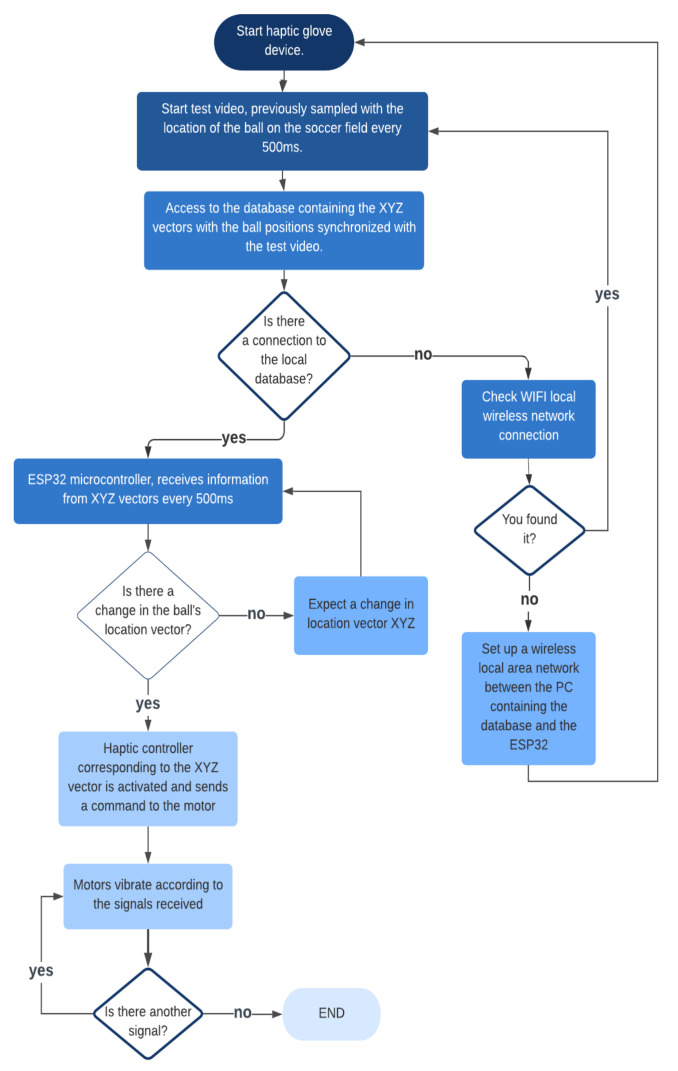
Flowchart of haptic glove operation.

In the flow chart in Figure 5, the operation of the haptic glove is shown.

A list of the materials used for the construction of the glove and their characteristics are given in Table 2.

**Figure 6:**
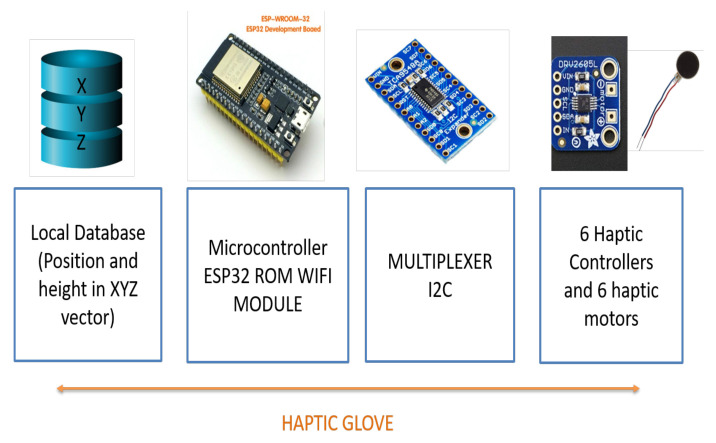
General diagram of the haptic glove parts.

Figure 6 shows a diagram of haptic glove general parts, basically, the software part is fed from data that is stored in a local database with XYZ vector information with the location of the ball sampled every 500 ms. The hardware part consists of an ESP32 ROM microcontroller with a WIFI module included, which will be responsible for receiving the data from the database through a TCP client-server connection, and at the same time coding and interpreting this information to send it through an Inter-Integrated Circuit (I2C) connection to the haptic controllers, which are the ones that send the vibration effects to the motors. However, as there are six motors to be handled, it was necessary to connect an I2C multiplexer to the microcontroller’s output. In this diagram, the device’s power supply is not detailed, but in the following section, the process of its implementation is detailed.

### 3.4. Validation Test

A small sample of users was chosen for the validation tests because of the difficulty meeting the test requirements and having the time required to carry out the test. The participation of 15 visually impaired people who wanted to collaborate with the test’s execution was achieved. Most of them were totally blind, and nobody could see the ball. They liked soccer and sports in general, two essential requirements to obtain statistical data and trends based on the use and experience of using the glove. This study’s main objective is to have a real appreciation of visually impaired people and validate the innovation and usefulness of the TV haptic glove. In order to obtain these results, a questionnaire survey was developed with mostly closed responses, where people had to rate their answers on a scale of 1 to 5, based on the Likert scale model [54].

## 4. Development and Implementation of the TV Haptic Glove

**Figure 7:**
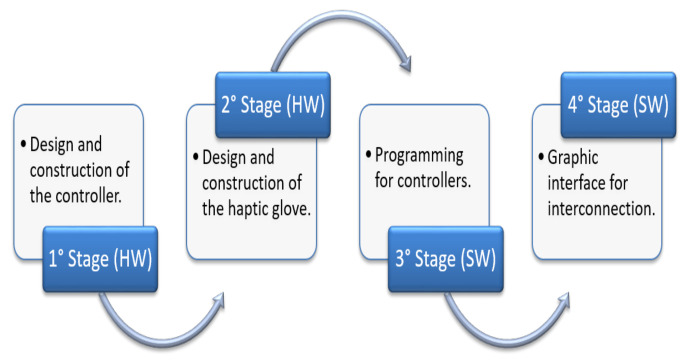
Diagram of the glove construction process.

Once the appropriate methodology for its operation and its construction materials had been defined, the device building process began. For the haptic glove, the implementation was divided into four stages, the first two in hardware and the next two in software, as shown in the diagram of Figure 7.

**Figure 8:**
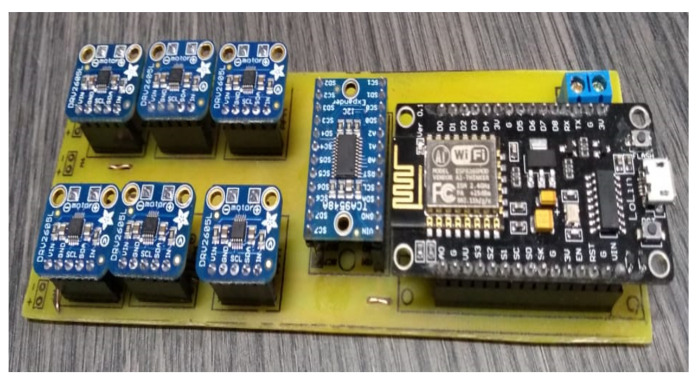
Haptic glove control board.

The first hardware stage was the controller’s design and construction inside a specific box that must be adapted to a bracelet to be carried on the user’s arm. For this, it was thought that it should be as small as possible in size and lightweight for the comfort of the person using it, as it would have to be worn for the duration of a sporting event. For the controller design, a board was designed and developed that integrates the ESP32 WIFI microcontroller, the I2C multiplexer, and the six haptic controllers, one for each engine. Figure 8 shows the implementation of the finished board.

**Figure 9:**
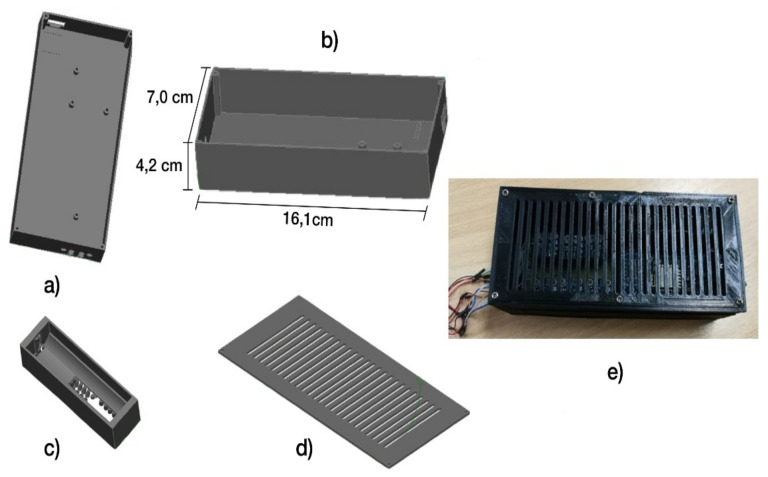
Haptic glove control board case.

A specific box was designed for this board, which allows it to be integrated with the energy part. It contains a lithium battery to give it the necessary autonomy to visualize a sports event and allows having a power charging interface to connect it to the power network through its universal serial bus (USB) port and not have problems with the power while the event lasts. Figure 9 shows the outline of the case design containing the drivers and the power part. (a) top view, (b) side view, (c) lithium battery container (d) case cover, and (e) final result top view of the case, which was made using a 3D printer.

**Figure 10:**
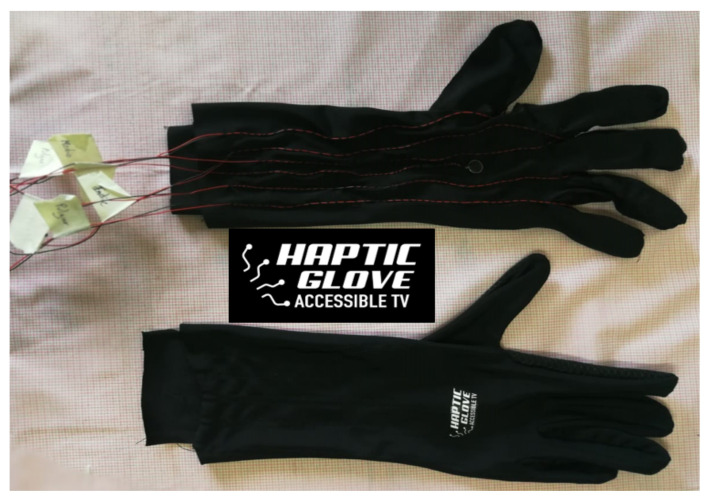
Glove making process and haptic device logo.

For the next hardware stage, the glove was designed and built-in one size from an elastic fabric material that fits various hand sizes. The characteristics of the material used for the glove’s construction are lightness and freshness to avoid excessive heat and sweating of the hand. The idea of making this glove from scratch was to mimic as much as possible the cables that carry energy and control to haptic motors in the points already mentioned in the previous section. Figure 10 shows glove-making process. Above the image, the glove’s internal pair with the cables and the haptic motors, and below the outer part of the glove with the haptic device’s logo.

The next stage, software, was the programming of the controller. This was done with an Arduino compiler based on C language programming. In this stage, the code that allows reading the information from a database and executing the haptic controllers’ actions with the different vibration effects in the motors was made. For this, it was necessary to load the different libraries from the main microcontroller manufacturers and the haptic controllers. This generates a code that is loaded into the memory of the ESP32 microcontroller. Here, the network’s name to be connected, the network access key, a fixed IP address, the gateway, and the broadcast address of the network are also indicated.

The fourth and last stage is also software. It was implemented to achieve communication between the haptic device and a graphic interface on the PC that allows to load a database in text form (Excel file) or perform the testing and training stage by entering a vector or loading sequences that generate various vibrations of the engines. The graphic interface was designed in a javascript that manages the sending of data through a predefined IP and port 80 by processing a web service connection. In this link, you can find all the project code https://github.com/dfvillamarin/hapticTV.git, accessed on 30 September 2020.

**Figure 11:**
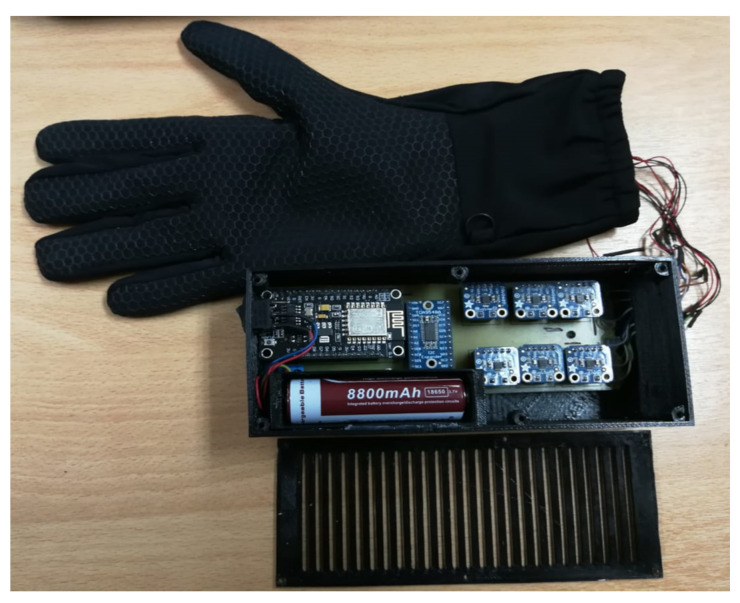
Haptic glove final product.

Finally, the integration of the controller with the glove and the graphic interface was carried out. The final hardware product is shown in Figure 11.

**Figure 12:**
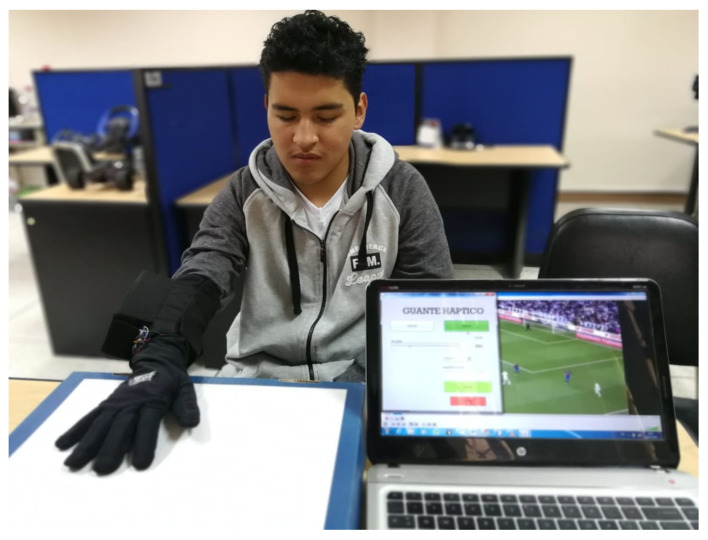
Haptic glove being used by a blind person.

To get an overview of the final result and how the haptic glove is used, you can see in Figure 12, a visually impaired person who is using the haptic glove. A bracelet is placed on his arm that fits the size of his forearm. This bracelet contains the device driver and connects to the glove, which in turn has mimicked cables and motors, so it is virtually unnoticeable to the end-user that he has cables and motors in his hand. The image also shows a PC, which is connected to the same WIFI network as the haptic device, to send the sequence of vibrations synchronized with the audio of the video of the sports meeting observed in the PC background.

## 5. Integration of the Haptic Glove with the Television

The main objective of this work is to validate the utility that the glove can have. However, without deviating from the objective, we want to propose at least three possible solutions to achieve the TV video’s integration with the vibrations generated by the haptic glove. The main challenge in this part is to achieve correct synchronization between the video sequence and the glove’s vibrations.

**Figure 13:**
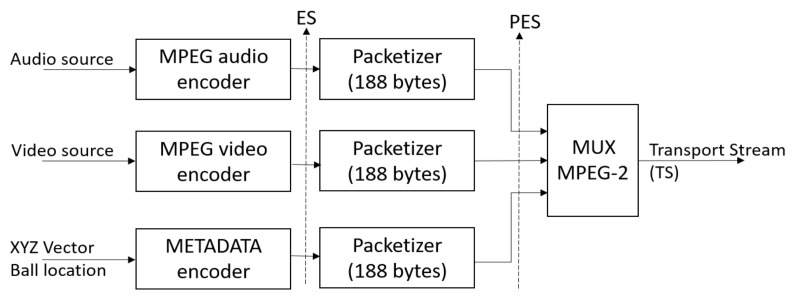
Conformation of the Transport Stream with Metadata.

The first solution would be to include the vector information with the ball location as metadata within the Transport Stream (TS). Using a special encoder, create an Elementary Stream (ES) that explicitly contains the vector information with the ball’s location. Assign a Packet Identifier (PID) for these data, package them into fixed 188 byte packets and then multiplex them along with the other audio, video, and data streams. A diagram of this process is shown in Figure 13.

The opposite process would occur at the receiver, where we would have to work on a special decoder that understands that metadata. It would identify the PID of the packets containing the ball location information and extract them in vector form so that later it could be integrated in some way with the WIFI network and the haptic controller. In Section 2 we presented some works that can serve as reference [46,47,48].

The second solution, and it is possible the easiest for its integration, would transmit this vector’s information with the ball’s location through the interactive platforms that have the digital television systems. For example, GINGA for ISDB-Tb (Integrated Services Digital Broadcasting–Terrestrial Built-in) or HbbTV (Hybrid broadcast broadband TV) for DVB-T (Digital Video Broadcasting–Terrestrial). For HbbTV, the integration already exists because there is always a web server connection. It could be updated with real-time information and stored in a database, even to access this information at any time. This would be a great advantage since the user could observe a sports meeting that is not live. Another advantage that brings the HbbTV 2.0.1. version is the CSS (Companion Screen Sync) protocol that allows synchronization with other monitoring devices, which would practically solve the integration with the haptic glove device [55].

Finally, we can propose an alternative approach for conventional video HTTP web servers setting up adaptive streaming media sources. It would be including the vibration info as metadata within the standardized MPEG-DASH events through a media presentation description (MPD) file.

The other challenge, and it is likely more complicated, is generating this vector automatically and in real-time. This could be done using different technologies such as computer vision, through stereo vision using the stadium’s cameras, through a network of microphones on the field [56]. It is installing a global positioning system (GPS), a radio frequency identification (RFID), a near-field communication (NFC), or other technologies inside the soccer ball and forming a network of sensors inside the field, among other alternatives. However, it remains a new line of research that could be proposed to analyze and determine which would be the most feasible and efficient. On Section 2 we mentioned two related works [49,50].

In order to fulfill our main objective of finding the usefulness of the haptic glove; initially as detailed in Section 3, the vectors of the location of the ball within the regions of the field were obtained using manual sampling. That is to say, visually the video of a soccer match was paused, and its location was written in the form of vectors within an editable text document (Excel). Later, this file was used as a local database and synchronized with the video also manually.

## 6. Analysis and Evaluation of Results

This section includes the methodology used to evaluate the results, the sample of participants, and a detailed analysis of the results obtained.

### 6.1. Methodology and Evaluation Parameters

For the analysis of the results, evaluation tests were carried out with the glove and the sample video on a group of 15 blind people: 12 men and 3 women, aged between 16 and 67. Users used the device for at least 30 min. Most did not use the glove for the 90 min of a soccer match because of their time availability. Explaining its operation and performing a previous stage of training took time and the time spent filling in the survey. The evaluation was not done for more than 30 min, so that the experience would not become too tired or dull and lose objectivity.

In order to compile and tabulate the results obtained in an orderly and precise way, surveys of questions were carried out, generally closed questions, where they had to choose from 1 to 5 according to the Likert scale [54], where 1 is very low, 2 low, 3 medium, 4 high and 5 very high. The survey was separated into two parts. The first part was carried out before using the glove. It consisted of collecting general information from the user and finding an initial perception of the prototype by the people who were going to use the glove.

**Figure 14:**
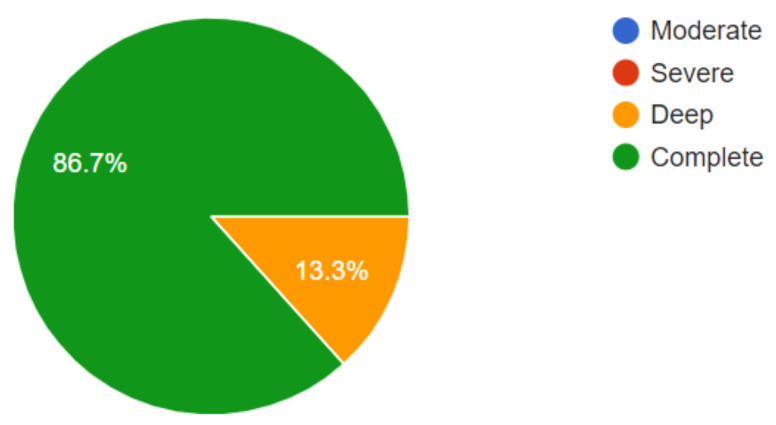
Percentage of visually impaired respondents.

The results in this first stage showed the respondents’ level of visual disability, which was very high, as shown in Figure 14.

Of the fifteen people surveyed, twelve participants had lost their sight for various reasons, such as accidents or illness, and three participants were born blind. It was a bit more complicated with these three participants to perform the tests because they had never seen a soccer field, although they were curious and expected to try the glove. Of the 15 respondents, 14 declared themselves to be right-handed and one ambidextrous, so they were optimal for the test, as the glove design was made for the right hand. The 15 respondents also stated that they had no previous experience with a technological device that allowed them to experience TV in an immersive way, which caused great expectations.

In terms of statistical analysis, two different ways are used. In the first one, the questions are analyzed individually on an ordinal Likert scale using descriptive statistics. To summarize the data numerically and visually, parameters such as mean, median, mode, standard deviation, symmetry, and kurtosis are used. In some questions, a bar graph is also shown to visualize the frequency of each item choice. The second way of analyzing the data uses other statistical tools such as bootstrap re-sampling and non-parametric statistical methods to obtain global results with confidence parameters. The non-parametric method used was the Mood’s medians test, based on a particular case of the Pearson’s chi-square test. While in the case of Bootstrap, it is still a parametric test that focused on the analysis of means and uses the t-tests to calculate confidence intervals. Table 3, Table 4 and Table 5 summarize the results of the application of these methodologies.

### 6.2. Initial Perception (before Doing the Test)

**Figure 15:**
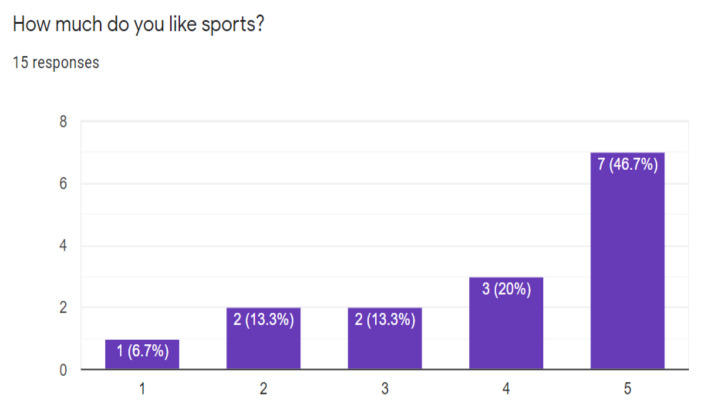
Respondents who enjoy sports.

**Figure 16:**
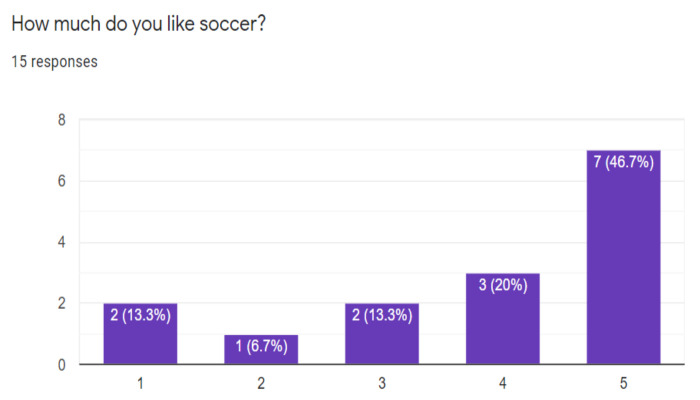
Respondents who enjoy soccer.

On some questions made to have an initial perception of the project, it was possible to know that the great majority if they liked the sports, especially soccer, even some had their favorite teams, Figure 15 and Figure 16 shows it.

**Figure 17:**
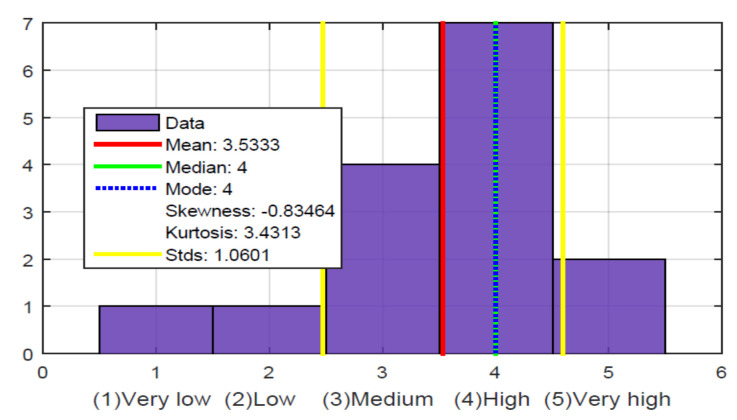
How much do you understand when you listen to a soccer commentator during a match transmission?

**Figure 18:**
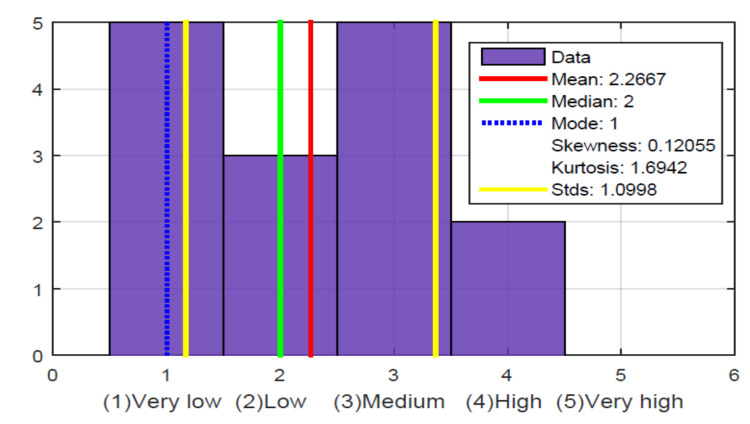
What level of perception do you have regarding the ball location on the soccer field while listening to the TV commentary?

**Figure 19:**
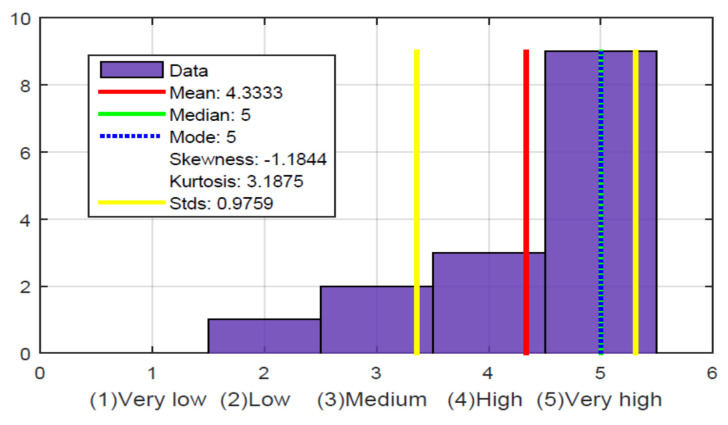
In your opinion, how much do the TV commercials create a distraction that makes you lose track of the play?

**Figure 20:**
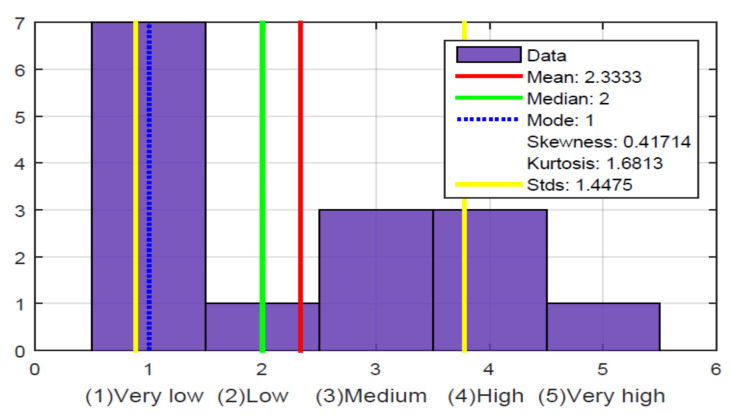
When a referee calls a side kick, what level of perception do you have about the place where the ball left the field?

**Figure 21:**
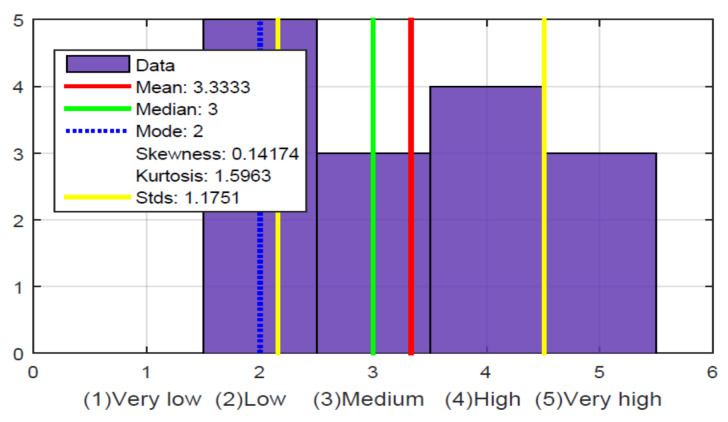
Do you know the team scores when a goal happens?

However, the most important questions were asked in this stage to know if a need exists and that the glove could provide a solution. Were the questions that are detailed in the Figure 17, Figure 18, Figure 19, Figure 20 and Figure 21.

From here on, the tabulated questions will be shown in the form of a histogram. Statistical values such as mean, median, mode, standard deviation, kurtosis value, and skewness will also be shown to better interpret the results obtained in the evaluation of the glove. Kurtosis is a statistical measure that determines the degree of concentration of a variable’s values around the central area of the frequency distribution. This will be positive and higher if there is a large concentration of values around its average. Skewness is a measure of symmetry, or more precisely, the lack of symmetry. A distribution, or data set, is symmetric if it looks the same to the left and right of the center point. As the data becomes more symmetrical, the value of its skewness approaches zero. Data with positive skewness or asymmetric to the right are so-called because the “tail” of the distribution points to the right and because the value of asymmetry is greater than 0. Data asymmetrical to the left or with negative skewness are so-called because the “tail” of the distribution points to the left and because the value of asymmetry is less than 0.

From these five questions, the hypothesis is validated that the level of perception they have of the ball’s location in a soccer game is low; in Figure 18, it is observed that the average and the median are in a low value. Although it could be the case that the narrator is explicit and says in which sector of the field the game is being played, which does not frequently happen in a TV soccer narrative. The soccer narrators who do it on RADIO are likely more explicit because they know that the listeners are not watching images of the game. In fact, at this stage of the survey, specifically with the question in Figure 17, it was possible to determine that some people, despite not knowing the ball’s location within the field, have a good perception of what happens in the sports match. For example, when there are commercials or the narrators stop their narration, they lose the match’s notion, which does not happen with people who typically attend the match because of the TV images.

Another problem that was identified is, for example, when they listen to team matches that they are not familiar with, since they do not know the name of their players, they lose interest since they hardly understand anything. In fact, when they hear a goal narrated, it is difficult for them to identify which team scored the goal precisely because they do not know the names of all the players. However, knowing the ball’s location would help understand what happens in the game if they know which side each team is located on.

### 6.3. Usability and User Experience Results

The second part of the survey was conducted when the glove users completed the tests, i.e., they used the haptic glove while listening to a soccer match. The questions were divided into three stages: the usability and user experience, the ergonomics and construction of the glove, and the requirements for future use and improvements to the device.

**Figure 22:**
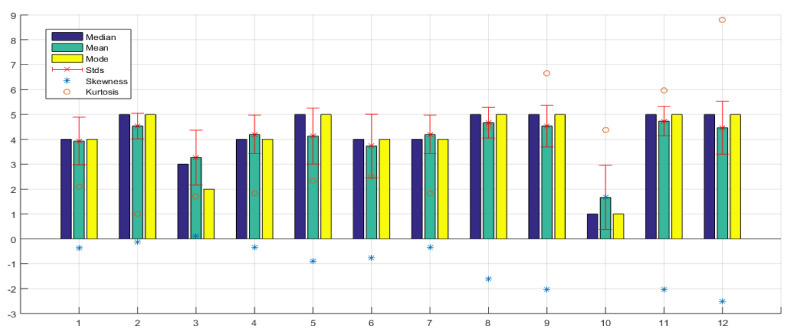
Usability and user experience evaluation results.

About the usability and user experience, Table 3 shows the 12 questions of this topic, with their respective statistical results. Figure 22 shows the statistical analysis of originally observed samples graphically.

Hereafter, in order to have a complete analysis of the data and overcome the limited number of samples obtained with the 15 blind people, we used a means Bootstrap resamples with a bilateral confidence interval of 95% and 1000 samples. We also used Mood’s median test as a second method. This technique tests the null hypothesis that the medians of populations from which two or more samples are drawn are identical also with a bilateral confidence interval of 95%. The data from each sample are assigned to two groups, one consisting of data whose values are above the two groups’ median, and the other consists of data whose values are at or below the median. A Pearson’s chi-square test is then used to determine whether the observed frequencies in each sample differ from the expected frequencies derived from a distribution combining the two groups.

In this first stage, the first question is the most important, as it validates that the glove’s objective was met, since a large percentage of people evaluated the glove positively and were able to locate the position of the soccer ball within the field.

**Figure 23:**
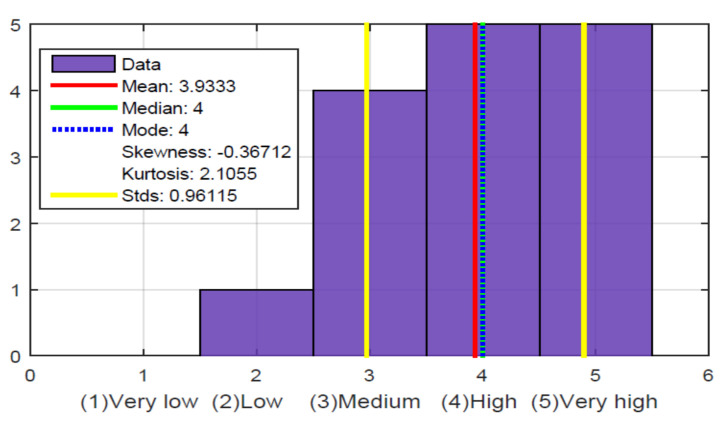
To what extent were you able to locate the soccer ball through the vibrations generated by the glove motors?

Even though this was the first time they used the glove and were not familiar with it, the results were positive, as seen in Figure 23. The average, median, and mode are in high values; 10 of the 15 respondents gave a high and very high rating, four people gave an average rating, and only one person gave a low rating. This person was the only one who felt burdened. The participant had been blind since birth and found it difficult to understand the methodology. In fact, he felt stressed by the glove. The following three questions validated in more detail which position was more comfortable for the users to recognize on the soccer field.

Figure 22, question 2, shows where they had the greatest ease in identifying the location of the ball, which was on the *X* axis, i.e., along the court. Question 3 shows where they had the greatest difficulty, which was identifying the ball in the *Y* axis, that is, along the width of the soccer field. This is because it was necessary to have an excellent perception and to be able to identify the three levels of vibration intensity. It is possible that this can be improved with training and more use of the haptic glove or by modifying the notification procedure for this parameter. Question 4 shows that the mean is also high concerning the location of the ball in the *Z* axis, that is, the height of the ball above the ground. It is lower than the difficulty involved in identifying the ball on the *X* axis, it is possible because some of them focused on identifying the XY plane, that is, the length and width of the soccer field, and the information on the height of the ball was not very relevant. Although it was useful to know this information for most, they were able to identify it easily.

About the level of intensity of the vibrations generated by the glove as shown in Figure 22, question 5, most think they are adequate. However, a small number of participants are likely more sensitive to touch, thinking that it could be modified to feel better with the glove.

Figure 22, question 6, shows that most participants perceive that there is an adequate synchronization between the narrative of the match and the location of the ball that they identified with the glove. Although in reality, there was a small gap that some people did manage to perceive. This small gap occurred mainly because the tests were performed on a 2.4 Ghz WiFi network, open and shared to replicate a real user scenario, where packet loss and delays due to channel congestion could occur. It is also important to mention that the hardware processing times could be controlled by software by adjusting the delays, but the adjustment was manual until proper synchronization was achieved. Another factor that could have generated synchronization errors was the fact that the test video sequences sampling was done manually so that some human error could have been generated.

Figure 22, question 7, reveals that the device, for the vast majority, was effortless to understand since they consider it intuitive. Taking into account that they had a brief explanation and training stage to perform the glove test. Figure 22, question 8, also reveals that the training stage was necessary where they identified the vibrations and became familiar with the glove.

**Figure 24:**
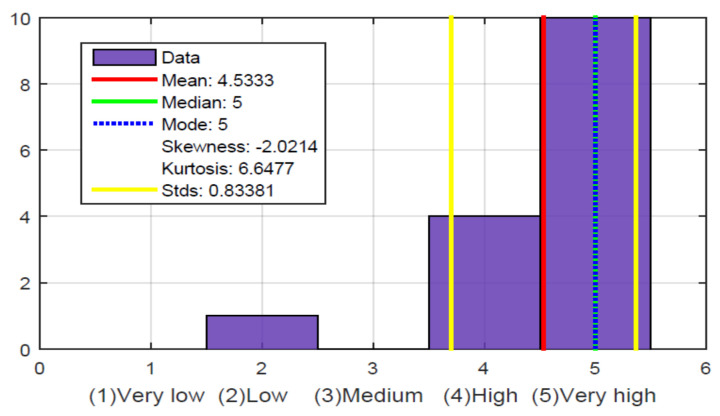
To what extent do you think the gadget is useful as an add-on for sports narration on TV?

Figure 22, question 9 and Figure 24, show the most crucial question validating the hypothesis that our work with the glove does have utility and provides a solution for these people who have been excluded and cannot enjoy this information on TV. Of the 15 people evaluated, 14 gave a high and very high evaluation. The only one gave a low rating, as he never felt comfortable with the glove and never really showed interest because he did not like sports, perhaps because of his condition, as he is a person who was blind from birth. The mean, median, and mode are at a very high value. The value of kurtosis also shows a high value because of the concentration of information at 5 (very high). The skewness value is negative since the mean is lower than the mode and the tail of the distribution is longer for values below the mean.

Additional questions were asked to determine the user’s satisfaction and experience, which are shown in Figure 22, questions 10, 11, and 12. It can be determined that most participants consider that it is not tiring to use the device, that they had an enjoyable experience, and that most would like to use the device again.

### 6.4. Ergonomic Results of Glove Construction

**Figure 25:**
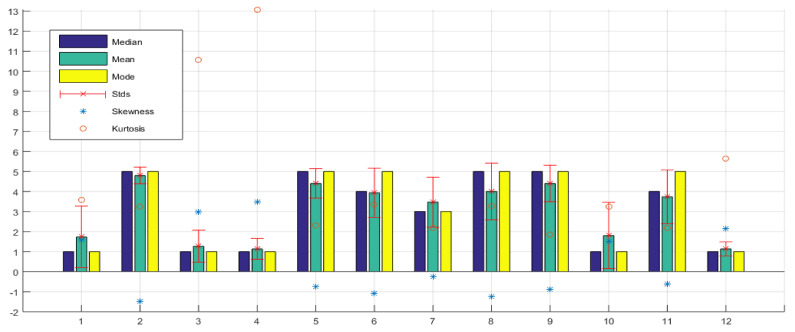
Ergonomics and construction of the glove evaluation results.

Regarding the ergonomics and construction of the glove Table 4 shows 12 questions with their respective statistical results. Figure 25, questions 1, 2, 3, 4, and 5, demonstrate that most respondents did not feel any discomfort when using the glove. Their perception of the glove’s quality was very high quality, both the glove and the bracelet did not generate heat or sweat, and in general, they felt comfortable using the glove.

In terms of the weight, as shown in Figure 25, question 6, most participants thought it is adequate, although some thought that the bracelet’s weight could be lowered. To give accurate data, the weight of the bracelet is 248 g. When examining the size, Figure 25, question 7, demonstrates more diverse results, with some participants believing that the bracelet’s size could be made smaller. Figure 9 shows the device size data. It is important to say that the battery and the entire board with the control circuit implemented are inside the bracelet. Regarding the size of the glove, as you can see in Figure 25, question 8, most of the participants fitted correctly with the glove, but other participants found it a little big, so they recommended that there should be at least three sizes and not just one size.

About the motors’ location, most participants found them to be right, although some participants recommended other points that could improve the design of the glove. Specifically, they recommended placing them so that the hand’s size does not matter. One option would be to change them instead of being in the fingertips, to place them in the proximal phalanx. Although we studied several points and the fingertips, we considered them the best option because they are the most sensitive points of the hand. Nevertheless, without a doubt, the option of doing more tests and determining a new point of the motors remain open. Figure 25, question 9, shows the satisfaction that users have about the location of the motors.

Figure 25, question 10, it is shown that the cables did not generate discomfort to most users. In fact, most of them did not even realize that the cables existed, as they were correctly mimicked within the glove’s construction.

Figure 25, question 11, it is shown that the vibration intensity of the motors generated different responses so that one could think of customizing the level of vibration intensity of the motors in an initial configuration.

Finally, in the last question concerning the ergonomics of the glove, the result of Figure 25, question 12, shows that there is no difficulty in wearing the glove on their hand. It also shows that they have very well-developed touch instincts, and just by feeling them, they can put it on quickly.

### 6.5. Glove Requirements for Future Use and Improvements

The last stage recorded was a survey that included questions to find future requirements and uses that can be given to the haptic device, Table 5 shows the questions and statistical results. However, we show the results that we consider most important in our analysis because if we take it into account, we could have a second version of the glove with new functions and considerable improvements.

**Figure 26:**
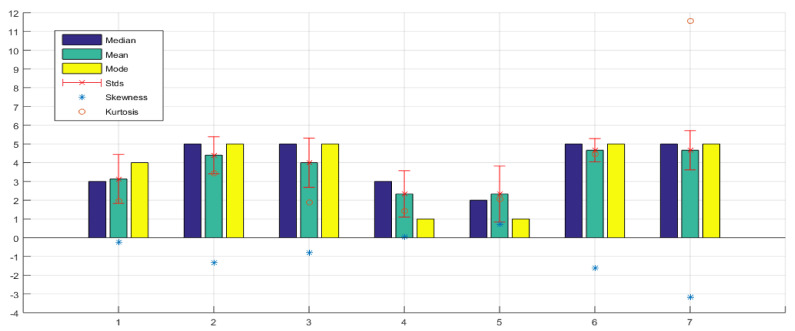
Evaluation results of requirements for future use and device improvements.

Figure 26, question 1, shows varied results of the level of perception that users have about the time that has elapsed in a soccer match. In fact, they were asked, during the course of the match, how long they think they are playing? Some people came very close to the game time, but others did not give a close answer. With this, it was determined the need for some mechanism to indicate the playing time. Since they manifested that they have problems in knowing the time of game in a soccer match, especially when they do not start to listen the game from the beginning. That they have to ask someone else to indicate the time that has passed on the television chronometer.

The same problem exists with the score of the game. Thus, they cannot watch the scoreboard that always remains visible on the television, and this can be seen in Figure 26, question 2, where most of the participants believe that the need to have an on-demand sound to indicate the score of the match is very high.

Figure 26, question 3, shows that when a goal occurs, most people are unaware of which team scored a goal. Additionally, when they are not familiar with the players’ names and the narrator does not indicate who possesses the ball, there is difficulty in identifying who scored the goal. Therefore, it may be a requirement that when a goal is scored, it needs to be indicated by the sound of which team scored it.

Figure 26, question 4, shows that there are a high number of people who indicate that when the referee whistles a foul on the field, they do not understand for whom the foul was whistled or who committed it. This is because they are generally not familiar with the names of the players heard in the narratives, which is shown in Figure 26, question 5. However, also there are soccer fans who have all the team’s player lists in their minds.

Figure 26, question 6, shows the need to increase buttons, gestures, and voice command in the glove, which, when carried out, will result in a response, e.g., time, score, match statistics, etc.

Figure 26, question 7, shows that there is great enthusiasm among participants to try the glove on other sports or applications. Other questions investigated which sports or applications they imagined could be applied to the glove, and the responses were varied. The other sports they mentioned were boxing, basketball, volleyball, tennis. It also occurred to them that it could be a useful device for them to play chess, electronic games, educational applications. They went further and proposed that the glove through vibrations could act as a guide for them to identify obstacles, potholes, stands, among others. Furthermore, since some of the participants play blind soccer, also known as blind football, they came up with the idea that the haptic device could guide them to play a soccer blind game. The main problem they face when playing soccer for the blind is that the fans scream or cheer for them, and that noise or ambient noise does not allow them to hear the bells that sound inside the balls they use. This makes it difficult to give continuity to the game.

In the last part of the survey, the participants were told about ten plays or situations in a soccer match. Focus on knowing which of these were more difficult for them to perceive and propose a solution with that information. Apart from the ball’s location on the field, this type of information can be sent through vibrations. The answers in this stage were varied since it also depends on the imagination and perception level that each person has. However, among the moves that are more difficult to perceive in a soccer game were: offside, dribbles or prepared moves, head out or in the area, fouls or infractions.

## 7. Discussion

The statistical data interpretation shows positive results that were analyzed in more detail in the analysis and evaluation of results section. The mean, mode, and median values in the three phases of the survey remained at high and very high values, except for those expected to have low and very low values as an ideal result. The standard deviation is acceptable and allows us to conclude that the procedure proposed for evaluating results was adequate. The values of kurtosis and skewness also helped us to understand better and interpret the results. In kurtosis, we have high and medium positive values that indicate an intense concentration of values in the central area of the distribution. In skewness, we have negative values in most cases, which means that the tail of its distribution is to the left, then it indicates that the greater concentration of values is in the scale of high and very high. Using the bootstrap method to better estimate the population size based on resampling of the original small sample helped us validate our statistical results. It was observed that the trend of the originally calculated means was maintained, and the variance was improved with a larger population considering a bilateral confidence interval of 95%, and this allows us to infer a true value of the mean parameter. It was also of great value to find a non-parametric method suitable for the small number of samples we had available and ordinal and non-quantitative data. The data we had were rather qualitative but based on a null hypothesis test, we can infer that the median results originally obtained were within those calculated with Mood’s median test, also with a 95% confidence interval. These results encourage us to continue with the work and to carry out the execution of future works.

After the analysis of the results, it is concluded that the glove proposal offers a real solution to the problem that blind people encounter when following a soccer match on TV. The glove’s evaluation and performance tests proved that it is really useful and satisfactory to use it for this vulnerable group, which is somehow excluded from this type of audiovisual application.

It can also be concluded that the haptic effect significantly improves the hearing experience that people with visual impairment live when attending sports programs on TV; the quality of experience felt by users was higher than when haptic feedback is not presented. It is worth discussing that, although the application was developed with a focus on delivering complementary information of the scenes that help the accessibility of blind people to the content of sporting events broadcast on television, whether they are live or pre-recorded in the case of VOD. With the feedback we received from the end-users, and based on their needs, it was determined that the glove gadget could also be used in the radio broadcast, in any sports video content regardless of the platform or transmission medium, even for physical events. For example, for visually impaired people attending a stadium or practicing soccer for the blind. To achieve this goal, we are currently working on finding a solution by applying artificial vision and deep learning techniques to automatically detect and track the ball automatically and generate the location coordinates.

The proposal was novel for the specific application used, but the evaluation process determined that it could be adapted and used in other applications in the future. For example, in other sports, even unrelated to the initial focus, we gave it in this immersive and interactive TV work. The haptic device’s construction in the form of a glove that fits the hand is feasible, was not obtrusive or intrusive to users, the bracelet fits perfectly on the forearm is comfortable and light. However, the size and weight could be improved in future work.

This work mentions at least three proposals to send this data and achieve integration with television’s broadcast system. Leaves open the research and analysis path to find the best solution to this challenge because the main limitation is not using it in a live soccer match or in real-time. It also leaves open broader research to determine the most appropriate technology to automate the generation of the ball location vector on the field.

## 8. Future Works

It is proposed to continue with the investigation to achieve a correct integration and synchronization with the television transmissions and carry out an adequate analysis of the most suitable technology to achieve the automatization of vectors generation with the location of the ball in real-time. Based on these investigations, it will be possible to continue with the solutions’ design and implementation stage. We are currently working on a more straightforward solution to solve the vector generation in real-time in an easy and fast way, although not completely automatic. In our next work, we will detail it together with the transmission of this information in the digital television platforms. In future work, we also want to achieve a second, much more interactive version of the glove with new functionalities, design improvements and mainly work on allowing the glove to be customizable to the user. Each user will control the intensity and duration of the vibrations according to their sensitivity and taste.

## Figures and Tables

**Table 1:** Detail of motor vibration.

Right Hand	Vibration Level		
	$Y_{1}$	$Y_{2}$	$Y_{3}$
Thumb $X_{1}$	Low	Medium	High
Index finger $X_{2}$	Low	Medium	High
Middle finger $X_{3}$	Low	Medium	High
Ring finger $X_{4}$	Low	Medium	High
Little finger $X_{5}$	Low	Medium	High
Back of the hand *Z*	$Z_{1}$ Null	$Z_{2}$ Medium	$Z_{3}$ High

**Table 2:** Materials list.

Qty.	Material/Model	More Details
1	ESP32 development board. [51]	Low-cost, low–power microcontroller with integrated Wi–Fi and Bluetooth dual–mode technology.
6	Haptic Controller Breakout RV2605L [52]	This module allows you to easily write Python code that controls the vibration of the motor. It can generate 123 vibrations effects.
6	Mini Motor Disc (ADA1201)	Vibrating Mini Motor Disc. DC3V/0.1 A
1	TCA9548A 1-to-8 I2C multiplexer [53]	A way to get up to 8 same–address I2C devices hooked up to one microcontroller. This multiplexer acts as a gatekeeper, shuttling the commands to the selected set of I2C pins with your command.
1	Lithium battery	DC5V, 8800 mAh
1	Micro USB 5 V module	For charging or powering the haptic device via the micro USB port.

**Table 3:** Usability questions, user experience questions and statistical results.

#	Question	Observed Samples (No. Samples = 15)			Bootstrap Samples for Mean (No. Re-Samples = 1000)			Non-Parametric Mood’s Median Test
		Mean	Median	Std Dev	Mean	Std Dev	95% Mean CI	95% Median CI
1	To what extent were you able to locate the soccer ball through the vibrations generated by the glove motors?	3.933	4	0.961	3.934	0.245	(3.467, 4.400)	(3, 5)
2	To what extent were you able to identify the ball location along the soccer field (across the longest dimension)?	4.533	5	0.516	4.530	0.132	(4.267, 4.800)	(4, 5)
3	To what extent were you able to identify the ball location across the soccer field (shortest field dimension)?	3.267	3	1.100	3.270	0.277	(2.733, 3.800)	(2, 4)
4	To what extent were you able to identify the ball height on the soccer field?	4.200	4	0.775	4.202	0.194	(3.800, 4.533)	(4, 5)
5	How suitable were the intensities of the vibrations to you?	4.133	5	1.126	4.134	0.280	(3.600, 4.667)	(3, 5)
6	According to you, was there a correct synchronization from the gadget between the commentary and the ball location?	3.733	4	1.280	3.752	0.321	(3.067, 4.400)	(3, 5)
7	To what extent do you think the gadget and its working are intuitive and easy to understand?	4.200	4	0.775	4.206	0.186	(3.867, 4.600)	(4, 5)
8	To what extent did you need a training stage?	4.667	5	0.617	4.658	0.155	(4.333, 4.933)	(4, 5)
9	To what extent do you think the gadget is useful as an add-on for sports narration on TV?	4.533	5	0.834	4.536	0.206	(4.067, 4.867)	(4, 5)
10	How tiring was it to use the glove?	1.667	1	1.291	1.659	0.3194	(1.133, 2.333)	(1, 2)
11	How nice was the experience using the gadget?	4.733	5	0.594	4.740	0.149	(4.400, 5.000)	(5, 5)
12	How much would you like to use the gadget again?	4.467	5	1.060	4.465	0.267	(3.867, 4.867)	(4, 5)

**Table 4:** Questions of ergonomics and construction of the glove with statistical results.

#	Question	Observed Samples (No. Samples = 15)			Bootstrap Samples for Mean (No. Re-Samples = 1000)			Non-Parametric Mood’s Median Test
		Mean	Median	Std Dev	Mean	Std Dev	95% Mean CI	95% Median CI
1	Did you feel any discomfort during or after wearing the glove?	1.733	1	1.533	1.746	0.391	(1.000, 2.600)	(1, 1)
2	What sensation did you have about the material quality?	4.800	5	0.414	4.803	0.102	(4.600, 5.000)	(5, 5)
3	To what extent did the glove material cause sweating or heat?	1.267	1	0.799	1.269	0.199	(1.000, 1.667)	(1, 1)
4	To what extent did the bracelet material cause sweating or heat?	1.133	1	0.516	1.134	0.132	(1.000, 1.400)	(1, 1)
5	To what extent do you think the gadget design is comfortable?	4.400	5	0.737	4.387	0.180	(4.000, 4.733)	(4, 5)
6	To what extent did you feel the gadget weight was fitting?	3.933	4	1.223	3.926	0.296	(3.333, 4.467)	(3, 5)
7	To what extent did you feel the gadget size was right?	3.467	3	1.246	3.478	0.308	(2.867, 4.067)	(3, 5)
8	To what extent did your hand fit to the glove size?	4.000	5	1.414	3.993	0.337	(3.267, 4.600)	(3, 5)
9	To what extent did you feel the motors were rightly located?	4.400	5	0.910	4.407	0.224	(3.933, 4.800)	(3, 5)
10	To what extent were the cables uncomfortable to you?	1.800	1	1.6562	1.776	0.415	(1.000, 2.600)	(1, 1)
11	How suitable do you think the intensity changes of the vibration motors were?	3.733	4	1.335	3.734	0.329	(3.067, 4.400)	(3, 5)
12	How difficult was it to put on the glove?	1.133	1	0.352	1.131	0.090	(1.000, 1.333)	(1, 1)

**Table 5:** Questions for requirements of future use and improvements to the device with statistical results.

#	Question	Observed Samples (No. Samples = 15)			Bootstrap Samples for Mean (No. Re-Samples = 1000)			Non-Parametric Mood’s Median Test
		Mean	Median	Std Dev	Mean	Std Dev	95% Mean CI	95% Median CI
1	About how much time has passed during a soccer match, what perception level do you have about the match duration?	3.133	3	1.302	3.134	0.327	(2.533, 3.800)	(2, 4)
2	To what extent would you like to have information about the scoreboard?	4.400	5	0.986	4.393	0.243	(3.933, 4.800)	(4, 5)
3	When a goal happens, how aware are you about which team scored?	4.000	5	1.309	4.002	0.331	(3.333, 4.600)	(3, 5)
4	When the referee gives a foul, how much do you understand who committed the foul?	2.333	3	1.234	2.335	0.302	(1.733, 2.933)	(1, 3)
5	To what extent are you able to identify to which team the player mentioned in the narration belongs?	2.333	2	1.496	2.337	0.367	(1.667, 3.133)	(1, 4)
6	To improve your experience with this glove, how much would you like it to incorporate buttons, gestures and voice commands?	4.667	5	0.617	4.669	0.150	(4.333, 4.933)	(4, 5)
7	To what extent would you like to try the glove for a different sport or application?	4.667	5	1.047	4.682	0.250	(4.133, 5.000)	(5, 5)

## Data Availability

The data presented in this study are available in https://n9.cl/9juct.
